# 845. Clinical Outcomes in Patients Treated with High vs. Standard Dose Liposomal Amphotericin B for Invasive Mucormycosis

**DOI:** 10.1093/ofid/ofad500.890

**Published:** 2023-11-27

**Authors:** Sarah Valiante, Ramy H Elshaboury, Tiffany Wu, Fabiola Reyes, Chloe Lahoud, Andres E Franceschi, Sophia Koo, Alyssa R Letourneau, Sarah P Hammond

**Affiliations:** Massachusetts General Hospital , Langhorne, Pennsylvania; Massachusetts General Hospital, Boston, Massachusetts; Massachusetts General Hospital, Boston, Massachusetts; Brigham and Women's Hospital, Boston, Massachusetts; Brigham and Women's Hospital, Boston, Massachusetts; Brigham and Women's Hospital, Boston, Massachusetts; Brigham and Women's Hospital, Dana-Farber Cancer Institute, Boston, Massachusetts; Massachusetts General Hospital, Boston, Massachusetts; Massachusetts General Hospital, Boston, Massachusetts

## Abstract

**Background:**

Mucormycosis is a life-threatening invasive fungal infection associated with high mortality. Amphotericin B is first-line therapy; however, the benefit of high doses remains uncertain. The purpose of this study is to describe outcomes in patients treated with high dose versus standard dose liposomal amphotericin B (L-AMB) for mucormycosis.

**Methods:**

This was a retrospective cohort study of adults ≥ 18 years treated with L-AMB for proven or probable invasive mucormycosis at Massachusetts General Hospital and Brigham & Women’s Hospital from 2016-2022. L-AMB dosing was based on clinician preference. Patients were excluded if L-AMB began > 7 days prior to admission. Patients were categorized per L-AMB dose: standard dose (all doses < 6 mg/kg) and high dose (≥ 6 mg/kg for at least one dose). The primary outcome was 6- and 12-week mortality.

**Results:**

67 patients were treated with L-AMB for proven or probable mucormycosis during the study period: 57 with standard dose and 10 with at least 1 high dose of L-AMB. Baseline characteristics are shown in Table-1. More patients in the standard dose group had underlying hematologic malignancy. About one third of patients had underlying diabetes and this was evenly distributed between groups. Table-2 shows infection- and treatment-related details. Proven invasive mucormycosis was diagnosed in 51/57 (89%) and 6/10 (60%) patients in the standard and high dose groups, respectively. The most common site of infection in both groups was skin/bone. Approximately 35/57 (61%) and 8/10 (80%) patients in the standard and high dose groups, respectively, had surgical debridement. Mortality at 6- and 12- weeks and renal toxicity outcomes are shown in Table-3. In the entire cohort at 6- and 12 weeks death occurred in 25/67 (37%) and 33/67 (49%) patients, respectively; no difference in mortality was seen between the standard and high dose groups. There appeared to be no difference in renal outcomes, though more patients in the standard dose group required renal replacement therapy within 6-weeks of diagnosis.

**Figure:**
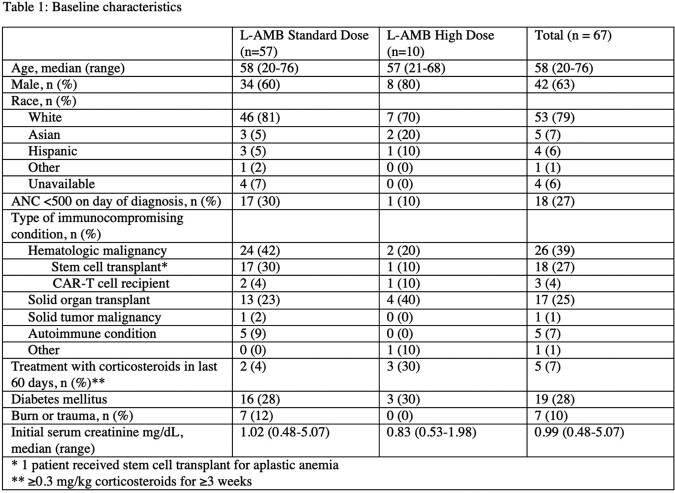

**Figure:**
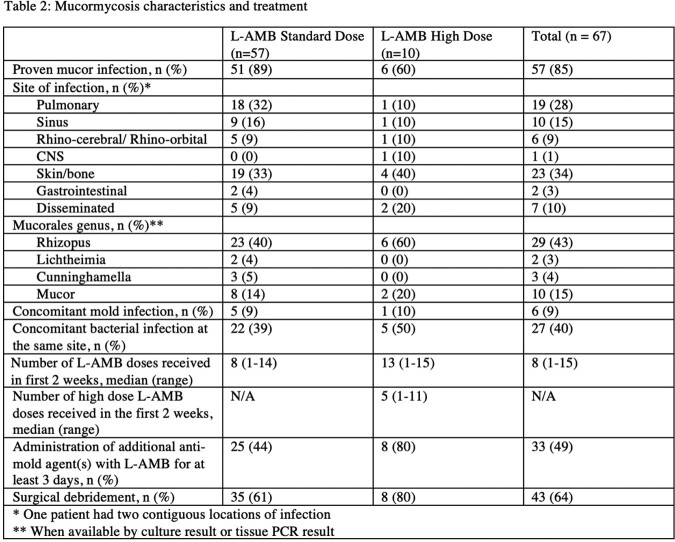

**Figure:**
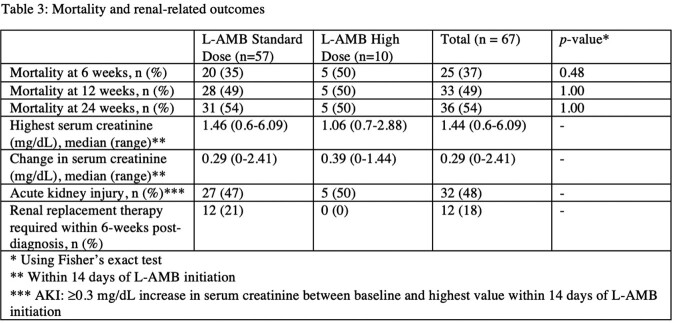

**Conclusion:**

We observed no difference in 6- and 12-week mortality among patients treated with L-AMB at standard and high doses for invasive mucormycosis. Mortality at 6- and 12-weeks of mucormycosis diagnosis remains high.

**Disclosures:**

**Ramy H. Elshaboury, PharmD**, DayZero Diagnostics: Advisor/Consultant|Gilead, Inc: Grant/Research Support|Thermofisher: Grant/Research Support **Sophia Koo, MD, SM**, Aerium Therapeutics: Advisor/Consultant|GSK: Grant/Research Support|Merck: Grant/Research Support|Scynexis: Grant/Research Support **Sarah P. Hammond, MD**, F2G: Advisor/Consultant|F2G: Grant/Research Support|GSK: Grant/Research Support|Pfizer: Advisor/Consultant|Scynexis: Grant/Research Support|Seres therapeutics: Advisor/Consultant

